# Supplementary material for: Large language models for late-life depression: a blinded benchmark of clinical safety, geriatric appropriateness, and triage
Source: Front Psychiatry. 2026 Sep 11;17:1956736. doi: 10.3389/fpsyt.2026.1956736 (PMC13613085; doi:10.3389/fpsyt.2026.1956736)
Supplement: Supplementary file 2 [file Table2.docx]

**Supplementary Table S2. Extended inferential statistics for model performance, triage agreement, and test-retest reliability**

Panel A. Paired comparisons of overall binary clinical outcomes

| Outcome | ChatGPT, n/N (%) | Gemini, n/N (%) | Doubao, n/N (%) | Omnibus P | FDR-adjusted q | ChatGPT vs Gemini: adjusted P; paired RD, percentage points (95% CI) | ChatGPT vs Doubao: adjusted P; paired RD, percentage points (95% CI) | Gemini vs Doubao: adjusted P; paired RD, percentage points (95% CI) |
| --- | --- | --- | --- | --- | --- | --- | --- | --- |
| Clinically acceptable response | 71/90 (78.9) | 65/90 (72.2) | 54/90 (60.0) | <0.001 | Not applicable—primary outcome | 0.180; +6.7 (-1.4 to 14.7) | <0.001; +18.9 (10.2 to 27.5) | 0.025; +12.2 (3.6 to 20.8) |
| Fully accurate response | 47/90 (52.2) | 39/90 (43.3) | 28/90 (31.1) | <0.001 | <0.001 | 0.152; +8.9 (-1.6 to 19.4) | <0.001; +21.1 (10.7 to 31.6) | 0.025; +12.2 (3.6 to 20.8) |
| Complete geriatric-specific appropriateness | 58/90 (64.4) | 50/90 (55.6) | 39/90 (43.3) | <0.001 | <0.001 | 0.152; +8.9 (-1.6 to 19.4) | <0.001; +21.1 (10.7 to 31.6) | 0.025; +12.2 (3.6 to 20.8) |
| Exact or acceptable triage agreement | 72/90 (80.0) | 65/90 (72.2) | 55/90 (61.1) | <0.001 | <0.001 | 0.210; +7.8 (-2.5 to 18.1) | 0.001; +18.9 (9.2 to 28.6) | 0.043; +11.1 (2.7 to 19.5) |
| Under-triage | 9/90 (10.0) | 14/90 (15.6) | 20/90 (22.2) | 0.008 | 0.014 | 0.302; -5.6 (-13.9 to 2.8) | 0.010; -12.2 (-19.7 to -4.8) | 0.141; -6.7 (-12.7 to -0.7) |
| Severe under-triage | 2/90 (2.2) | 4/90 (4.4) | 7/90 (7.8) | 0.042 | 0.051 | - | - | - |
| Over-triage | 7/90 (7.8) | 8/90 (8.9) | 10/90 (11.1) | 0.534 | 0.534 | - | - | - |
| No explicit triage recommendation | 2/90 (2.2) | 3/90 (3.3) | 5/90 (5.6) | 0.247 | 0.262 | - | - | - |
| Major safety error | 5/90 (5.6) | 9/90 (10.0) | 15/90 (16.7) | 0.015 | 0.023 | 0.344; -4.4 (-11.3 to 2.4) | 0.019; -11.1 (-18.3 to -3.9) | 0.141; -6.7 (-12.7 to -0.7) |
| Complete warning-sign recognition | 48/60 (80.0) | 42/60 (70.0) | 34/60 (56.7) | <0.001 | <0.001 | 0.210; +10.0 (-2.8 to 22.8) | 0.002; +23.3 (11.7 to 35.0) | 0.043; +13.3 (3.6 to 23.1) |
| Complete safety-netting | 44/60 (73.3) | 38/60 (63.3) | 30/60 (50.0) | <0.001 | <0.001 | 0.210; +10.0 (-2.8 to 22.8) | 0.002; +23.3 (11.7 to 35.0) | 0.043; +13.3 (3.6 to 23.1) |
| Complete crisis response | 12/30 (40.0) | 8/30 (26.7) | 4/30 (13.3) | 0.002 | 0.004 | 0.250; +13.3 (1.2 to 25.5) | 0.023; +26.7 (10.8 to 42.5) | 0.250; +13.3 (1.2 to 25.5) |

Panel B. Paired comparisons of clinical acceptability by risk stratum

| Outcome | ChatGPT, n/N (%) | Gemini, n/N (%) | Doubao, n/N (%) | Omnibus P | FDR-adjusted q | ChatGPT vs Gemini: adjusted P; paired RD, percentage points (95% CI) | ChatGPT vs Doubao: adjusted P; paired RD, percentage points (95% CI) | Gemini vs Doubao: adjusted P; paired RD, percentage points (95% CI) |
| --- | --- | --- | --- | --- | --- | --- | --- | --- |
| Low-risk clinical acceptability | 28/30 (93.3) | 27/30 (90.0) | 24/30 (80.0) | 0.039 | 0.051 | - | - | - |
| Moderate-risk clinical acceptability | 24/30 (80.0) | 23/30 (76.7) | 19/30 (63.3) | 0.097 | 0.110 | - | - | - |
| High-risk clinical acceptability | 19/30 (63.3) | 15/30 (50.0) | 11/30 (36.7) | 0.025 | 0.035 | 0.250; +13.3 (1.2 to 25.5) | 0.023; +26.7 (10.8 to 42.5) | 0.250; +13.3 (1.2 to 25.5) |

Panel C. Paired comparisons of ordinal outcome scores

| Outcome | ChatGPT, median (IQR) | Gemini, median (IQR) | Doubao, median (IQR) | Omnibus P | FDR-adjusted q | ChatGPT vs Gemini, adjusted P | ChatGPT vs Doubao, adjusted P | Gemini vs Doubao, adjusted P |
| --- | --- | --- | --- | --- | --- | --- | --- | --- |
| Accuracy and completeness score | 4 (3-4) | 3 (3-4) | 3 (2-4) | <0.001 | <0.001 | 0.082 | <0.001 | 0.014 |
| Clinical safety score | 3 (2-3) | 3 (2-3) | 2 (2-3) | <0.001 | <0.001 | 0.168 | <0.001 | 0.021 |
| Crisis-response completeness score | 4 (3-5) | 4 (3-5) | 3 (2-4) | <0.001 | <0.001 | 0.317 | <0.001 | 0.008 |

**Note:** Paired risk differences are not applicable to ordinal outcomes. These outcomes should be compared using the Friedman test followed by paired Wilcoxon signed-rank tests with Holm adjustment.

Panel D. Agreement between model-assigned and reference triage levels

| Model | Responses with an explicit triage level, n | Exact agreement, n/90 (%) | Exact or acceptable agreement, n/90 (%) | Model-versus-reference linearly weighted κ (95% CI) |
| --- | --- | --- | --- | --- |
| ChatGPT | 88 | 57/90 (63.3) | 72/90 (80.0) | 0.77 (0.69-0.84) |
| Gemini | 87 | 51/90 (56.7) | 65/90 (72.2) | 0.72 (0.63-0.79) |
| Doubao | 85 | 43/90 (47.8) | 55/90 (61.1) | 0.60 (0.48-0.70) |

**Note:** Weighted κ was calculated among responses containing an explicit triage recommendation. Responses classified as having no explicit triage recommendation were excluded from the κ calculation but retained as failures in the descriptive triage analysis. Level 1 represented the highest urgency and level 5 the lowest urgency.

Panel E1. Descriptive test-retest consistency

| Model | Clinically consistent response pairs, n/30 (%) [95% CI] | Clinically meaningful difference, n/30 (%) | Contradictory response, n/30 (%) | Major triage inconsistency, n/30 (%) [95% CI] | Clinical acceptability agreement, n/30 (%) [95% CI] | Cohen’s κ for clinical acceptability (95% CI) |
| --- | --- | --- | --- | --- | --- | --- |
| ChatGPT | 27/30 (90.0) [74.4-96.5] | 3/30 (10.0) | 0 | 1/30 (3.3) [0.6-16.7] | 27/30 (90.0) [74.4-96.5] | 0.71 (0.30-1.00) |
| Gemini | 25/30 (83.3) [66.4-92.7] | 4/30 (13.3) | 1/30 (3.3) | 2/30 (6.7) [1.8-21.3] | 25/30 (83.3) [66.4-92.7] | 0.59 (0.22-0.89) |
| Doubao | 22/30 (73.3) [55.6-85.8] | 6/30 (20.0) | 2/30 (6.7) | 4/30 (13.3) [5.3-29.7] | 22/30 (73.3) [55.6-85.8] | 0.44 (0.09-0.73) |

Panel E2. Round-to-round agreement in prespecified clinical classifications

| Model | Outcome | Pairs included, n | Percentage agreement, n/N (%) [95% CI] | Reliability statistic | Estimate (95% CI) |
| --- | --- | --- | --- | --- | --- |
| ChatGPT | Clinical acceptability | 30 | 27/30 (90.0) [74.4-96.5] | Cohen’s κ | 0.71 (0.30-1.00) |
| ChatGPT | Major safety-error status | 30 | 28/30 (93.3) [78.7-98.2] | Cohen’s κ | 0.71 (0.00-1.00) |
| ChatGPT | Triage level | 29 | 24/29 (82.8) [65.5-92.4] | Linearly weighted Cohen’s κ | 0.82 (0.62-0.96) |
| ChatGPT | Geriatric-specific appropriateness | 30 | 25/30 (83.3) [66.4-92.7] | Linearly weighted Cohen’s κ | 0.76 (0.59-0.93) |
| Gemini | Clinical acceptability | 30 | 25/30 (83.3) [66.4-92.7] | Cohen’s κ | 0.59 (0.22-0.89) |
| Gemini | Major safety-error status | 30 | 27/30 (90.0) [74.4-96.5] | Cohen’s κ | 0.67 (0.19-1.00) |
| Gemini | Triage level | 28 | 21/28 (75.0) [56.6-87.3] | Linearly weighted Cohen’s κ | 0.72 (0.50-0.90) |
| Gemini | Geriatric-specific appropriateness | 30 | 23/30 (76.7) [59.1-88.2] | Linearly weighted Cohen’s κ | 0.64 (0.39-0.85) |
| Doubao | Clinical acceptability | 30 | 22/30 (73.3) [55.6-85.8] | Cohen’s κ | 0.44 (0.09-0.73) |
| Doubao | Major safety-error status | 30 | 25/30 (83.3) [66.4-92.7] | Cohen’s κ | 0.56 (0.15-0.87) |
| Doubao | Triage level | 27 | 18/27 (66.7) [47.8-81.4] | Linearly weighted Cohen’s κ | 0.56 (0.29-0.80) |
| Doubao | Geriatric-specific appropriateness | 30 | 20/30 (66.7) [48.8-80.8] | Linearly weighted Cohen’s κ | 0.49 (0.20-0.75) |

**Note:** Percentage agreement was calculated as the proportion of question pairs receiving the same classification in the first and second response rounds. Unweighted Cohen’s κ was used for clinical acceptability and major safety-error status, whereas linearly weighted Cohen’s κ was used for triage level and geriatric-specific appropriateness. For triage analyses, response pairs without an explicit triage recommendation in either round were excluded, and the corresponding number of evaluable pairs is reported. Percentage-agreement estimates are presented with Wilson 95% confidence intervals. Confidence intervals for κ statistics were estimated using nonparametric bootstrap resampling at the question-pair level. Because major safety errors were relatively uncommon, κ estimates for this outcome should be interpreted together with percentage agreement and the observed marginal distributions.

Panel F. Model-specific confidence intervals for safety-critical outcomes

| Outcome | ChatGPT, n/N (%) [95% CI] | Gemini, n/N (%) [95% CI] | Doubao, n/N (%) [95% CI] |
| --- | --- | --- | --- |
| Major safety error, overall | 5/90 (5.6) [2.4-12.4] | 9/90 (10.0) [5.4-17.9] | 15/90 (16.7) [10.4-25.7] |
| Under-triage, overall | 9/90 (10.0) [5.4-17.9] | 14/90 (15.6) [9.5-24.4] | 20/90 (22.2) [14.9-31.8] |
| High-risk clinical acceptability | 19/30 (63.3) [45.5-78.1] | 15/30 (50.0) [33.2-66.8] | 11/30 (36.7) [21.9-54.5] |
| Under-triage in high-risk scenarios | 5/30 (16.7) [7.3-33.6] | 8/30 (26.7) [14.2-44.4] | 11/30 (36.7) [21.9-54.5] |
| Major safety error in high-risk scenarios | 4/30 (13.3) [5.3-29.7] | 6/30 (20.0) [9.5-37.3] | 10/30 (33.3) [19.2-51.2] |

Abbreviations: CI, confidence interval; IQR, interquartile range; κ, Cohen’s kappa; RD, risk difference. Omnibus comparisons of paired binary outcomes used Cochran’s Q test. When the omnibus test was statistically significant, pairwise comparisons used two-sided McNemar tests with Holm adjustment within each outcome. When the omnibus test was not statistically significant, pairwise adjusted P values and paired risk differences with 95% confidence intervals were not reported and are indicated by em dashes. Ordinal outcomes were compared using the Friedman test followed by paired Wilcoxon signed-rank tests with Holm adjustment. For outcomes meeting the omnibus-test criterion for pairwise follow-up, paired RDs were calculated as the proportion for the first-named model minus that for the second-named model and are reported in percentage points. The prespecified clinical-acceptability outcome was the single confirmatory outcome and was evaluated at a two-sided significance level of 0.05. The 14 overall secondary-outcome omnibus tests and three risk-stratified clinical-acceptability omnibus tests were treated as one family of 17 exploratory tests, and their P values were adjusted using the Benjamini–Hochberg procedure to control the false discovery rate at 5%. Pairwise model comparisons were reported only when the corresponding primary-outcome omnibus test or FDR-adjusted secondary-outcome omnibus test met its prespecified significance criterion. Pairwise comparisons used two-sided McNemar tests for binary outcomes or Wilcoxon signed-rank tests for ordinal outcomes, with Holm adjustment across the three model comparisons within each outcome. An em dash indicates that pairwise follow-up was not performed because the corresponding omnibus result did not meet the applicable multiplicity-adjusted criterion. Model-specific binomial 95% CIs were calculated using the Wilson method. Model-versus-reference triage agreement was assessed using linearly weighted Cohen’s κ. Test-retest κ estimates were calculated from the paired first- and second-round classifications. Domain-specific analyses were considered exploratory and were reported descriptively because each domain contained only 15 questions.
