# Supplementary material for: Large language models for late-life depression: a blinded benchmark of clinical safety, geriatric appropriateness, and triage
Source: Front Psychiatry. 2026 Sep 11;17:1956736. doi: 10.3389/fpsyt.2026.1956736 (PMC13613085; doi:10.3389/fpsyt.2026.1956736)
Supplement: Supplementary file 3 [file Table3.docx]

Supplementary Table S3. Component decomposition and sensitivity analyses of the clinical-acceptability outcome

Panel A. Responses meeting each component of the prespecified clinical-acceptability definition

| Component criterion met | ChatGPT, n/N (%) | Gemini, n/N (%) | Doubao, n/N (%) |
| --- | --- | --- | --- |
| Accuracy and completeness score ≥3 | 82/90 (91.1) | 78/90 (86.7) | 69/90 (76.7) |
| Clinical safety score ≥2 | 84/90 (93.3) | 80/90 (88.9) | 72/90 (80.0) |
| No major safety error | 85/90 (94.4) | 81/90 (90.0) | 75/90 (83.3) |
| No under-triage | 81/90 (90.0) | 76/90 (84.4) | 70/90 (77.8) |
| All action-critical warning signs recognized | 48/60 (80.0) | 42/60 (70.0) | 34/60 (56.7) |
| All action-critical geriatric factors recognized | 78/90 (86.7) | 72/90 (80.0) | 63/90 (70.0) |
| All applicable criteria met: prespecified clinical acceptability | 71/90 (78.9) | 65/90 (72.2) | 54/90 (60.0) |

Panel B. Sensitivity analyses using alternative acceptability definitions

| Outcome definition | ChatGPT, n/N (%) | Gemini, n/N (%) | Doubao, n/N (%) | Omnibus P | ChatGPT vs Gemini, adjusted P | ChatGPT vs Doubao, adjusted P | Gemini vs Doubao, adjusted P |
| --- | --- | --- | --- | --- | --- | --- | --- |
| Core-safety definition | 79/90 (87.8) | 73/90 (81.1) | 62/90 (68.9) | <0.001 | 0.146 | 0.001 | 0.025 |
| Prespecified clinical-acceptability definition | 71/90 (78.9) | 65/90 (72.2) | 54/90 (60.0) | <0.001 | 0.180 | <0.001 | 0.025 |
| Stringent optimal-response definition | 42/90 (46.7) | 34/90 (37.8) | 23/90 (25.6) | <0.001 | 0.152 | 0.002 | 0.038 |

Panel C. Number of failed component criteria among responses not meeting the prespecified definition

| Failure pattern | ChatGPT | Gemini | Doubao |
| --- | --- | --- | --- |
| Responses not clinically acceptable, n | 19 | 25 | 36 |
| Exactly one failed criterion, n/N (%) | 5/19 (26.3) | 6/25 (24.0) | 6/36 (16.7) |
| Two or more failed criteria, n/N (%) | 14/19 (73.7) | 19/25 (76.0) | 30/36 (83.3) |

Note: Component criteria in Panel A are not mutually exclusive. Warning-sign recognition was applicable to 60 questions; criteria designated as not applicable were not counted as failures in the clinical-acceptability classification. The action-critical geriatric-factor criterion required recognition of the factors necessary to determine the correct interpretation, urgency, and immediate action and was therefore narrower than the separately reported category of complete geriatric-specific appropriateness. The core-safety definition required an accuracy and completeness score ≥3, clinical safety score ≥2, no major safety error, and no under-triage. The stringent optimal-response definition required an accuracy and completeness score of 4, clinical safety score of 3, no major safety error, exact or acceptable triage agreement, complete warning-sign recognition when applicable, and complete geriatric-specific appropriateness. Omnibus comparisons used Cochran’s Q test. Statistically significant omnibus results were followed by pairwise McNemar tests with Holm adjustment. The sensitivity analyses were post hoc and were used to evaluate the robustness of model ranking rather than as additional confirmatory tests.
